# Investigation of Contacts of a Health Care Worker Who Worked While Ill with Pertussis — Maryland, August–September 2014

**Published:** 2015-01-23

**Authors:** Kasi M. Chu, Lucas A. Johnson

**Affiliations:** 1 Department of Preventive Medicine and Biometrics, Uniformed Services University of the Health Sciences, Bethesda, Maryland

On September 5, 2014, the public health department of a Maryland hospital was notified of a case of *Bordetella pertussis* infection confirmed by polymerase chain reaction (PCR) in a staff health care worker (HCW). The HCW experienced onset of diarrhea and malaise (nonrespiratory symptoms atypical of the catarrhal phase of pertussis) on August 26. By September 2, paroxysms of coughing led the HCW to consult a colleague, who ordered the PCR test, prescribed a 5-day course of azithromycin, and advised avoidance of patient care until treatment completion. Contrary to the hospital’s infection control policy, neither the HCW nor the colleague reported the presumptive diagnosis of pertussis to the hospital’s public health department. The HCW continued to work in the outpatient department until the positive PCR result was received on September 5, at which time the hospital’s public health department was first notified. The hospital barred the HCW from further work at the hospital while ill, and, in collaboration with local and state public health counterparts, began a contact investigation and stratified patient and HCW contacts by level of exposure.

The HCW had received tetanus, diphtheria, and acellular pertussis vaccine (Tdap) in 2010 and reported an ill family member who had been exposed at school during a widespread outbreak of pertussis affecting the surrounding community in August (1). In all, 47 persons were identified as being exposed to the HCW, including 31 patients ranging in age from 7 days to 12 years (six of whom were too young to receive diphtheria, tetanus, and acellular pertussis vaccine [DTaP]) and 15 HCWs. Of these exposed persons, 22 were considered high-risk contacts (seven patients and 15 HCWs) because they were aged <12 months, or contacts who themselves have close contact with infants under 12 months, pregnant women, or persons with preexisting health conditions at risk for severe illness or complications from pertussis. All 22 high-risk contacts were assessed for symptoms and received postexposure prophylaxis according to established guidelines (2). Additionally, six HCW high-risk contacts (three staff physicians, two residents, and one nurse) reported symptoms suggestive of pertussis and were excluded from work until completion of a course of antibiotics. No patient contacts reported symptoms. Nasopharyngeal swabs obtained from all symptomatic high-risk contacts were negative for pertussis by PCR. The remaining low-risk contacts, or their identified parents or guardians, were screened for symptoms, informed of the low-risk nature of the exposure, and provided education on the signs and symptoms of pertussis. All 47 persons identified as exposed were contacted by public health investigators.

HCW presenteeism (i.e., working while sick) can jeopardize the well-being of patients and coworkers (3). Because of the need to investigate and limit exposures, clinical activities in a facility can be disrupted when staff members are potentially exposed to transmissible disease. HCWs should not work while ill with a potentially contagious condition.
